# Biochemical and molecular identification of lactic acid bacteria and yeasts with technological qualities from ogi (a cereal-based fermented food in Nigeria)

**DOI:** 10.5114/bta/214383

**Published:** 2026-03-31

**Authors:** Praise Temilade Ozabor, Taiwo Samson Olumakinde, Timilehin Emmanuel Oluokun, Johnson Olaleye Oladele, Ilesanmi Festus Fadahunsi

**Affiliations:** 1 Department of Microbiology, Osun State University, Osogbo, Osun State, Nigeria; 2 Food Microbiology and Biotechnology Unit, Department of Microbiology, University of Ibadan, Oyo State, Nigeria; 3 Multidisciplinary Research Laboratory, Osun State University, Osogbo, Nigeria; 4 Veterinary Physiology and Pharmacology Unit, College of Veterinary Medicine and Biomedical Sciences, Texas A&M University, College Station, Texas, United States of America; 5 Phytochemistry and Toxicology Unit, Royal Scientific Research Institute, Osun State, Nigeria

**Keywords:** cereal fermentation, starter cultures, polymerase chain reaction (PCR), lactic acid, diacetyl, hydrogen peroxide

## Abstract

**Background:**

This study aimed to isolate, characterize, and identify lactic acid bacteria (LAB) and yeasts with technological qualities from spontaneously fermented cereal gruels traditionally known as *ogi*.

**Materials and methods:**

LAB and yeasts were isolated and screened for pathogenicity tests using standard methods. LAB and yeasts were subjected to biochemical characterization with API 50 CHL and API 20C AUX, respectively. Molecular identification was conducted using 16S rRNA gene sequencing, ITS 4 & 5 for DNA amplification, polymerase chain reaction, gel electrophoresis, and Sanger sequencing. The technological qualities of the identified LAB and yeasts were assessed using established protocols.

**Results:**

The API-based characterization of the nonpathogenic organisms revealed that the LAB mainly comprised *Lactobacillus* and *Lactococcus* genera, while *Candida, Cryptococcus*, and *Trichosporon* were the prominent yeast genera. However, the results for molecular identification and percentage of occurrence showed that the LAB involved in the fermentation process mainly included *Lactobacillus* (70%) and *Lactococcus* (30%), while *Candida* (40%), *Cryptococcus* (35%), *Derbaryomyces* (10%), *Trichomonascus* (10%), and *Naganishia* (5%) were the main yeast genera. Additionally, *Trichomonascus ciferri* was identified as a novel organism isolated from *ogi*. Highest concentrations of lactic acid, hydrogen peroxide, and diacetyl were recorded in *ogi* containing *Lactobacillus delbrueckii* RSL11 and *Lactobacillus* sp. MLL5, with values of 30.0 ± 0.01 mg/g, 38.0 ± 0.00 g/l, and 46.0 ± 0.001 g/l, respectively.

**Conclusion:**

Taken together, LAB and yeasts are important microorganisms with technological potentials for use as starter cultures in food fermentation processes for producing safe and functional foods.

## Introduction

Nigerian indigenous fermented foods and beverages represent a significant cultural heritage among the Nigerian people and across Saharan and sub-Saharan African countries (Johansen et al. 2019; Jeong et al. 2022). Fermented food products serve as dietary staples, refreshments, and complementary foods for young children, adults, and the elderly (Gabaza et al. 2019). In recent times, there has been an increase in the consumption of indigenously fermented foods as they are perceived by consumers to provide human health benefits, have organoleptic properties and nutritional values, and have extended shelf-life qualities (Marco et al. 2017; Motlhanka et al. 2018; Banwo et al. 2022). In Nigeria, *ogi*, typically made from millet, maize, and sorghum, is a popularly consumed fermented cereal gruel as it is readily available and affordable and has a relatively high content of essential macro and micronutrients (Beyene et al. 2020).

Previous studies have shown that thorough characterization of the microorganism of interest is a prerequisite for selecting appropriate starter cultures. Lactic acid bacteria (LAB) and yeasts have been reported as the two most important microbial species involved in the spontaneous fermentation of cereal grains (Gabaza et al. 2017; Gabaza et al. 2019; Otunba et al. 2021). Additionally, the use of functional microbial starter cultures during fermentation processes is vital for producing health-promoting, stable, and consistent food products that can help eliminate or reduce specific diseases and disease conditions and for reducing inconsistency in fermented foods (Todorov et al. 2017).

Babarinde et al. (2019), Fadahunsi et al. (2020), and Chourasia et al. (2022) reported that LAB can produce antagonistic primary and secondary inhibitory metabolites such as organic acids, hydrogen peroxide, carbon dioxide, and enzymes beneficial for human health, while yeasts convert small molecules of sugars into ethanol, carbon dioxide, and flavor-enhancing compounds.

Yeasts play various roles in food fermentation, including stimulation of LAB, fermentation of carbohydrates, production of flavor compounds, degradation of cyanogenic glycosides and mycotoxins, and production of tissue-degrading enzymes (Tamang et al. 2016; Olojede et al. 2020). Furthermore, fermentation has long been known to enhance the activities of indigenous microorganisms, improve the bioavailability and mobilization of inherent metabolites and bioactive compounds that can ameliorate oxidative stress-associated diseases, improve organoleptic properties of the food, and increase the shelf life of fermented foods (Adesulu-Dahunsi et al. 2019; Adebo and Medina-Meza 2020; Fadahunsi et al. 2020; Banwo et al. 2021; Mengesha et al. 2022).

Although microorganisms can be identified and characterized using conventional techniques, recent studies have revealed that these techniques are less sensitive and exhibit poor reliability (Liu et al. 2021). Hence, molecular identification is the preferred method as it provides more tangible and specific understanding of the microbial ecology of fermented food products compared to conventional techniques (Assohoun-Djeni et al. 2016; Otunba et al. 2021). Moreover, the consumption of functional cereal-based fermented foods has been linked with an increase in microbiota diversity (Ofosu et al. 2023).

In the present study, LAB and yeasts were isolated from spontaneously fermented *ogi* (a typical fermented gruel consumed in Nigeria and other sub-Saharan African countries) prepared using four different cereal substrates. The isolated organisms were subjected to pathogenicity tests for hemolysis, DNase, and gelatin liquefaction and characterized and identified using API kits and molecular methods; additionally, their technological properties related to amylase, lactic acid, diacetyl, and hydrogen peroxide production were assayed. The present study aimed to select nonpathogenic LAB and yeasts with technological qualities as starter cultures for the fermentation of microbiologically safe, functional food products/nutraceuticals.

## Materials and methods

### Laboratory preparation of ogi

Cereal grains, namely maize (*Zea mays*), millet (*Pennisetum glaucum*), and sorghum (*Sorghum bicolor*), were purchased from a local market in Osogbo, Osun State, Nigeria. The grains were validated by the Plant Biology Department of Osun State University, Osogbo. All samples were packaged inside zip-lock bags and transported to the microbiology laboratory of Osun State University for further processing. The samples were then cleaned by winnowing and handpicking dirt, broken grains, and stones; soaked in clean water for 48 h; wet-milled; sieved; and allowed to ferment spontaneously for 72 h on the laboratory bench as described by Ozabor et al. (2022).

### Serial dilution

One gram (1 g) of each portioned fermented slurry was 10-fold serially diluted in sterile test tubes. The slurries were dissolved in 9 ml of sterile distilled water for stock preparation. Each stock solution was aseptically dispensed into 9 test tubes, which were placed in test-tube racks. Stock solutions diluted to 10^3^ and 10^7^ dilution factors were plated on sterile agar plates (Fadahunsi et al. 2020; Ojokoh et al. 2020).

### Isolation of LAB and yeasts

LAB and yeasts were isolated using deMan, Rogosa, and Sharpe (MRS) agar (HiMedia Laboratories, Kennett Square, USA) and yeast extract (YEA) agar (HiMedia Laboratories), respectively, with the pour plate culturing technique. The cultured MRS agar plates were incubated at 37^o^C for 24–48 h under anaerobic conditions, while YEA plates were incubated aerobically at 30^o^C for 48–72 h. To obtain pure cultures, representative distinct colonies were repeatedly subcultured and stored on MRS and YEA slants. Pure stock cultures were kept at 4^o^C in a refrigerator for further analysis (Fadahunsi and Olubodun 2021; Banwo et al. 2022).

### Pathogenicity tests for isolated LAB and yeast species

#### Hemolysis tests

Hemolysis is defined as the breakdown of red blood cells. This test was conducted to determine the ability of the isolated LAB and yeast species to produce hemolysin, an enzyme that lyses red blood cells. Briefly, 24-h-old MRS and YEA broth cultures of LAB and yeasts, respectively, were aseptically streaked onto blood agar plates. The inoculated Petri dishes were incubated anaerobically at 37^o^C for 24–48 h for LAB and aerobically at 30^o^C for 48–72 h for yeasts (Fadahunsi and Olubodun 2021). The plates were observed for alpha, beta, and gamma hemolysis.

#### DNase test

The DNase test was performed to determine the ability of LAB and yeast isolates to produce DNase, an enzyme mediating DNA degradation. A loopful of 24-h-old broth cultures of LAB and yeasts in MRS and YEA, respectively, was aseptically streaked on sterile DNase agar plates containing methyl green as an indicator. The inoculated Petri dishes were incubated anaerobically at 37^o^C for 24–48 h for LAB and aerobically at 30^o^C for 48–72 h for yeasts (Fadahunsi and Olubodun 2021).

### Gelatin liquefaction

Briefly, 24-h-old broth cultures of LAB and yeasts were inoculated into sterile gelatin medium supplemented with 10% MRS and YEA agar media, respectively, in 100 ml Erlenmeyer flasks. The inoculated plates were incubated anaerobically at 37^o^C for 3–5 days for LAB and aerobically at 30^o^C for 5–7 days for yeasts. The cultured plates were observed for gelatin liquefaction (Fadahunsi and Olubodun 2021).

### Sugar fermentation tests for the isolated LAB and yeasts

Ten nonpathogenic representative LAB isolates were phenotypically characterized with reference to *Bergey’s Manual of Determinative Bacteriology* (9^th^ edition, 2000), while 25 nonpathogenic yeast isolates were phenotypically characterized using the fungi compendium (Alexopolus). Sugar fermentation tests for the selected LAB and yeast isolates were conducted using the analytical profile index (API) kits, namely API 50 CHL (Biomerieux, France) and API 20C AUX (Biomerieux), respectively, in accordance with the manufacturer’s instructions.

### Extraction of bacterial and fungal genomic DNA

Nonpathogenic LAB and yeast isolates were subjected to molecular characterization. The genomic DNA of pure representative bacterial and fungal colonies was extracted using the Quick-DNA^TM^ Miniprep Plus Kit (Zymo Research). Overnight grown cultures of LAB and yeasts from MRS and YEA broths (200 ml), respectively, were aseptically transferred into microcentrifuge tubes. Next, approximately 200 ml of biofluid and cell buffer supplemented with 20 ml proteinase K was added to the tubes. The reaction mixtures were vortexed for 10–15 s and incubated at 55^o^C for 10 min. One volume of genomic binding buffer was added to the reaction mixture and vortexed for 10–15 s.

The mixtures were transferred from the microcentrifuge tubes into a Zymo-Spin^TM^ IIC-XLR column placed inside collection tubes. The mixtures were centrifuged at 16,000 × g for 1 min. The collection tubes were discarded with the filtrates. A 400 ml DNA pre-wash buffer was added to the spin column placed inside the new collection tubes and centrifuged at 16,000 × g for 1 min. These collection tubes were also discarded. Next, 700 ml g-DNA wash buffer was added to the spin column and centrifuged at 16,000 × g for 1 min, and the collection tubes with the flow filtrates were discarded. Subsequently, 200 ml g-DNA wash buffer was added to the spin column and centrifuged at 16,000 × g for 1 min. The collection tube was discarded, and the spin column content was transferred into new microcentrifuge tubes. Approximately 50–55 ml DNA elution buffer was added to the reaction mixture and incubated for 5 min at room temperature on the laboratory bench. The reaction mixtures were centrifuged at ≥ 16,000 × g for 1 min to elute the DNA. The eluted DNA was stored at –20^o^C for further analysis. The obtained DNA was quantified using the NanoDrop spectrophotometer (Thermo Scientific, model no: WI 53711) before subjecting to polymerase chain reaction (PCR) analysis.

### PCR amplification of 16S rRNA and 18S rRNA and sequencing

The concentration and purity of the extracted DNA were quantified (A_260_/A_280_) using the NanoDrop spectrophotometer (Thermo Scientific, model no: WI 53711) at 280 and 230 wavelengths. The 16S rRNA region for LAB and the ITS region of large nucleotide subunits of 18S rRNA gene sequences of yeast isolates were amplified by PCR using the Master cycler (Nexus gradient; model no: 6331GQ015744) (Angelov et al. 2017; Tilahun et al. 2018; Banwo et al. 2021). The primers and PCR protocols used are presented in Tables 1 and 2. The LAB PCR products (amplicons) were visualized in 1% agarose gel stained with SYBR Safe DNA agarose gel stain and 1-kb ladder as a molecular ladder. Both LAB and yeast amplicons were sequenced at Inqaba Biotec West African Limited, Ibadan, Oyo State. The nucleotide sequences were subsequently submitted to the National Center for Biotechnology Information (NCBI) GenBank database and assigned accession numbers.

**Table 1:** Primers used for lactic acid bacteria (LAB) and yeasts polymerase chain reaction analysis

S/no.	Microorganism group	Primers	No. of nucleotides	Primer sequence 5’-3’ orientation	References
1	LAB	Lb-F	19	5’-GAGTTTGATCCTGGCTCAG-3’	Angelov et al. 2017; Tilahun et al. 2018; Banwo et al. 2021
		Lb-R	20	5’-AGAAAGGAGGTGATCCAGCC-3’	Angelov et al. 2017; Tilahun et al. 2018; Banwo et al. 2021
2	Yeasts	ITS4	20	5’-TCCTCCGCTTATTGATATGC-3’	Angelov et al. 2017; Tilahun et al. 2018; Banwo et al. 2021
		ITS5	22	5’GGAAGTAAAAGTCGTAACAAGG-3’	

**Table 2:** Polymerase chain reaction protocol for lactic acid bacteria (30 cycles) and yeasts (35 cycles)

Primers	Initial denaturationw	30/35 cycles	Final extension	Initial elongation	Final elongation
		Denaturation	Hybridization		
Lb-F Lb-R	95°C for 3 min	94°C for 30 s	50°C for 30 s	72°C for 90 s	72°C for 5 min
ITS4-F ITS5-R	95°C for 10 min	94°C for 30 s	55°C for 30 s	72°C for 1 min	72°C for 7 min

The PCR protocol was as follows: (i) initial denaturation, (ii) denaturation, (iii) hybridization, (iv) initial elongation, and (v) final elongation; the primers used for LAB and yeast isolates are presented in Table 2.

### Screening for the production of amylase, lactic acid, diacetyl, and hydrogen peroxide by the identified LAB and yeast isolates

#### Screening for amylase production

LAB and yeast isolates were screened for amylase production by using sterile starch agar plates. Petri dishes inoculated with LAB and yeast isolates were incubated anaerobically at 37^o^C for 24–48 h and aerobically at 30^o^C for 48–72 h, respectively. The plates were flooded with Lugol’s iodine and kept on the laboratory bench for 10–15 min. The appearance of clear halos around the streaked lines was considered positive (Banwo et al. 2021).

#### Screening for lactic acid production

Sodium hydroxide (NaOH, 0.1N) was titrated against 25 ml of 24-h-old broth cultures of LAB by using 3 drops of phenolphthalein as the indicator. NaOH addition was continued until the color changed. LAB and yeasts are the known predominant microbial species during fermentation processes. Hence, they are usually employed as starters for controlled fermentation (Birmeta et al. 2019; Onipede et al. 2021; Banwo et al. 2022; Ozabor et al. 2022). In the present study, LAB and yeasts dominated the fermentation of different cereal substrates. Besides cereal fermentations, LAB and yeasts are also predominant species in diary, meat, and other noncereal-related fermentation processes (Angelov et al. 2017; Akinyemi e01007t al. 2022). According to Taha et al. (2017) and Ashokbhai et al. (2022), fermentation increases the bioactivity and bioavailability of microorganisms in fermenting matrices. This implies that the different LAB and yeast isolates obtained in this study may be due to differences in the nutritional composition of cereal substrates, temperature, air supply, and ability of the fermenting organisms to adapt to fermenting matrices and pH. This observation is consistent with the earlier report of Mengesha et al. (2022), indicating that the occurrence of LAB and yeasts during fermentation can be attributed to the abovementioned parameters. Each milliliter of NaOH is equivalent to 90.08 mg of lactic acid. The same method was also used to assay lactic acid production in yeast isolates (Otunba et al. 2021).

#### Screening for diacetyl production

Briefly, 25 ml of 24-h-old broth cultures of LAB were aseptically dispensed separately into 100 ml Erlenmeyer flasks. Next, 7.5 ml of hydroxylamine solution was used for residual titration. Titrations were conducted with 0.1N HCl until a greenish endpoint was reached using bromophenol blue as the indicator. The equivalent factor of HCl to diacetyl is 21.5 mg. The same method was used for yeast isolates (Otunba et al. 2021).

#### Screening for hydrogen peroxide production

Briefly, 25 ml of diluted sulfuric acid was added to 100 ml Erlenmeyer flasks containing 25 ml of 24-h-old broth cultures of LAB. Titration was performed using 0.1N potassium permanganate, wherein 1 ml of 0.1N potassium permanganate is equivalent to 1.070 mg of hydrogen peroxide. Broth decolorization was recorded as the endpoint. The same method was also used to assay hydrogen peroxide production by the yeast isolates (Otunba et al. 2021).

### Statistical analysis

Evolutionary distances were computed using the Maximum Composite Likelihood method. Lactic acid, hydrogen peroxide, and diacetyl production values are expressed as mean ± SD of triplicate values.

## Results

### Microbial load and morphological characteristics of isolated yeasts and LAB from spontaneously fermented maize, sorghum, and millet

The highest LAB count (2.6 × 10^-3^ CFU/ml) was obtained for the spontaneously fermented maize *ogi*, while the spontaneously fermented sorghum *ogi* showed the lowest LAB count (4.0 × 10^–7^ CFU/ml). All LAB isolates were Gram-positive and catalase-negative. The cell shape ranged from round to irregular, while the cell size ranged from 0.4 to 1.0 mm. The colonies had smooth and rough surfaces and flat to raised elevation, were either white or cream in color, and had margins ranging from entire to lobate and undulate.

The highest (1.6 × 10^–3^ CFU/ml) and lowest (2.0 × 10^–7^ CFU/ml) yeast counts were obtained from the spontaneusly fermented maize and sorghum *ogi*, respectively. The yeast isolates showed oval cell shape; the colonies had smooth and rough surfaces and flat to raised elevation, were either white or cream in color, and had margins ranging from entire to undulate and lobate.

### Pathogenicity of yeasts and LAB isolates

All LAB and yeast isolates tested negative for DNase and gelatin liquefaction tests, while 87% of the LAB isolates and 89% of the yeast isolates tested negative for hemolysis test. Based on these results, only the organisms that tested negative in the pathogenicity tests were used for further analyses.

### Biochemical characterization of yeast and LAB isolates using API 20C AUX and API 50 CHL, respectively

The sugar fermentation tests for LAB in API 50 CHL revealed two major LAB genera, namely *Lactococcus* and *Lactobacillus*, while the sugar fermentation tests for yeasts in API 20C AUX showed 3 major yeast genera (*Candida, Cryptococcus*, and *Trichosporon*).

### Molecular identification and occurrence of API-characterized yeast and LAB isolates

**Figure 1:**
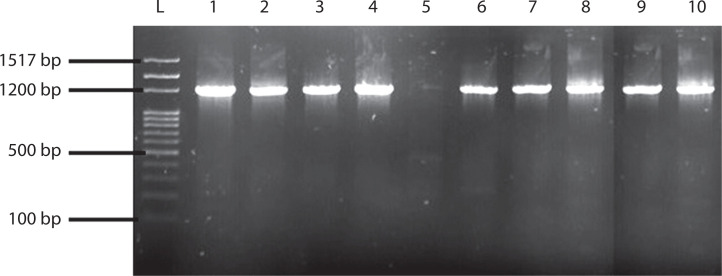
Agarose gel image of 10 selected lactic acid bacteria analyzed using specific primers Lb F and R, showing bands at 1200 bp. L = DNA molecular ladder

**Figure 2:**
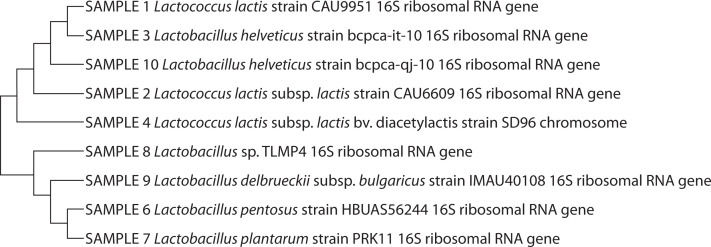
Phylogenetic tree of sequenced lactic acid bacteria amplicons

**Figure 3:**
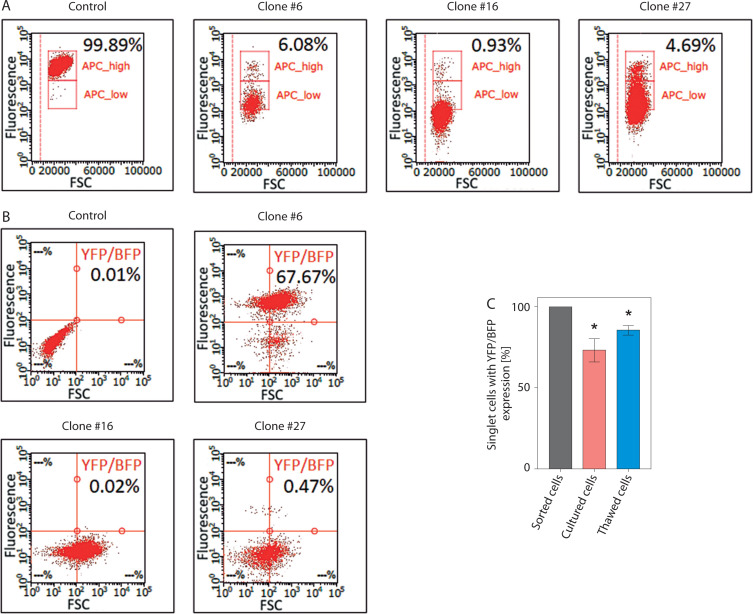
Agarose gel image of 25 selected yeasts analyzed using specific primers ITS4 and ITS5, showing bands between 300 and 600 bp. L = DNA molecular ladder

**Figure 4:**
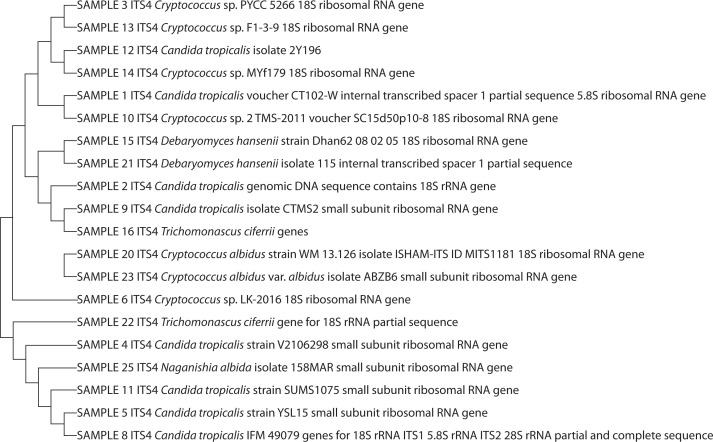
Phylogenetic tree of sequenced yeast amplicons

The API 50 CHL-characterized LAB isolates were molecularly identified as *Lactococcus lactis* (30%), *Lactobacillus helveticus* (20%), *Lactobacillus pentosus* (20%), *Lactobacillus* sp. (10%), *Lactobacillus plantarum* (10%), and *Lactobacillus delbrueckii* (10%). *L. lactis* (30%) had the highest percentage of occurrence (30%), while the lowest percentage of occurrence (10%) was recorded for *L. delbrueckii, L. plantarum*, and *Lactobacillus* sp. (Figures 1 and 2). The yeast isolates were molecularly identified as *Candida tropicalis* (36%), *Cryptococcus* sp. (28%), *Cryptococcus albidus* (12%), *Trichomonascus ciferri* (8%), *Candida ciferri* (4%), *Debaryomyces hansenii* (4%), *Naganishia albida* (4%), and *Cryptococcus laurentii* (4%). *C. tropicalis* had the highest percentage of occurrence (36%), while the lowest percentage of occurrence (4%) was recorded for *D. hansenii, N. albida*, and *C. laurentii* (Figures 3 and 4).

### Designation of the NCBI accession numbers

Some of the designated NCBI accession numbers in this study are as follows: *L. lactis* MZL1 (PP115580), *L. lactis* MZL4 (PP115581), *L. helveticus* MZL12 (PP115582), *L. lactis* MLL1 (PP115583), *L. pentosus* WSL5 (PP115584), *L. plantarum* RSL1 (PP115585), *Lactobacillus* sp. MLL8 (PP115586), *L. delbrueckii* RSL11 (PP115587), *L. helveticus* MZL17 (PP115588), *C. tropicalis* RSY43 (PP110435), *Cryptococcus* sp. MLY1 (PP110436), *C. tropicalis* MZY18 (PP110437), *Cryptococcus* sp. RSY48 (PP110438), *Cryptococcus* sp. MLY5 (PP110439), *C. albidus* RSY51 (PP110440), and *N. albida* WSY40 (PP110441).

All LAB and yeast isolates were amylase-positive and could synthesize lactic acid, diacetyl, and hydrogen peroxide. Among the LAB isolates, the highest and lowest lactic acid production levels (mg/g) were 30.0 ± 0.01 and 23.5 ± 0.005 for *L. delbrueckii* RSL11 and *L. lactis* MLL1, respectively. The highest and lowest hydrogen peroxide (g/l) production levels were 38.0 ± 0.00 and 24.0 ± 0.00 for *Lactobacillus* sp. MLL5 and *L. lactis* MZL4, respectively. The highest and lowest diacetyl production levels (g/l) were 46.0 ± 0.001 and 23.5 ± 0.005 for *Lactobacillus* sp. MLL5 and *L. lactis* MZL1, respectively.

Among the yeast isolates, *C. albidus* MZY30 and *N. albida* WSY40 produced the highest amount of lactic acid (3.25 ± 0.005 mg/g), while *C. tropicalis* WSY31 and *D. hansenii* WSY36 produced the lowest amount (1.95 ± 0.005 mg/g). The highest and lowest hydrogen peroxide production levels (g/l) were 34.0 ± 0.00 and 16.0 ± 0.00 for *C. ciferri* MLY11 and *C. tropicalis* RSY43, respectively. The highest and lowest diacetyl production levels (g/l) were 44.0 ± 0.001 and 18.5 ± 0.005 for *C. albidus* MZY30 and *Cryptococcus* sp. MLY1, respectively.

## Discussion

This study provides information on the indigenous LAB and yeast strains with technological qualities isolated, characterized, and identified from spontaneously fermented cereal gruels consumed in Nigeria. Compared to yeasts, LAB showed higher counts in all fermented grains during fermentation processes. This result may be attributed to the disparity in the nutritional composition of cereal grains used to prepare *ogi* and the pH and temperature of the fermenting matrix. This finding is consistent with earlier reports of Liu et al. (2021) and Banwo et al. (2022) for fermented home-made sauerkraut from three provinces in southwest China, wherein *Lactobacillus, Pediococcus*, and *Pichia* genera were identified, while *Lactobacillus* and *Candida* genera were identified in fermented sorghum gruels from Nigeria. For the past several decades, nonpathogenic microorganisms have been preferred as starter cultures for fermented food products. Hence, in the present study, the isolated LAB and yeast strains were screened for DNase and hemolysin production and gelatin liquefaction. The results revealed that the selected LAB and yeast strains were nonpathogenic. This finding aligns with the reports of Setta et al. (2022) and Banwo et al. (2022), who recommend that only food-grade microorganisms must be employed as starter cultures.

LAB and yeasts are reported to be the dominant microorganisms during fermentation processes and therefore are usually employed as starter cultures for controlled fermentation (Birmeta et al. 2019; Onipede et al. 2021; Banwo et al. 2022; Ozabor et al. 2022). In the present study, LAB and yeasts were isolated, characterized, and identified from different fermented cereal substrates. Besides cereal fermentation, LAB and yeasts have been found to be the predominant microbial species in dairy, meat, and other noncereal-related fermentation processes (Angelov et al. 2017; Akinyemi et al. 2022). According to Taha et al. (2017) and Ashokbhai et al. (2022), fermentation increases the bioactivity and bioavailability of microorganisms in fermenting matrices. This implies that variations in LAB and yeast strains identified in this study might be due to differences in the nutritional composition of cereal substrates, temperature, air supply, ability of the fermenting organism to adapt to the fermenting matrices, and pH. This observation agrees with the earlier report of Mengesha et al. (2022), which suggested that the occurrence of LAB and yeasts during fermentation is influenced by the abovementioned parameters.

*Lactococcus* and *Lactobacillus* are among the major LAB involved in cereal fermentation (Birmeta et al. 2019; Ozabor et al. 2022). *Saccharomyces cerevisiae, Candida tropicalis, Kluyveromyces marxianus*, and *Cryptococccus* sp. have been isolated, characterized, and identified in cereal grains (Ayodeji et al. 2021; Onipede et al. 2021). However, based on literature review, there is limited information on the isolation of *Naganishia* sp. and *Debaryomyces* sp. from fermented cereals, as reported in the present study. Amrouche et al. (2019) reported the presence of *Naganishia, Filobasidium, Malassezia, Mrakia, Rhodotorula*, and *Yarrowia* genera in fermented dromedary camel milk in Algeria. Joeng et al. (2022) also documented the isolation of *Debaryomyces* sp. in fermented sausages from Korea; this finding aligns with the observations of the present study. Furthermore, in the present study, *T. ciferri* was isolated from *ogi* for the first time. As a novel organism isolated from *ogi, T. ciferri* may be used as a potential starter culture for food fermentation processes after screening for technological and/or functional properties, including production of useful metabolites (diacetyl, hydrogen peroxide, ethanol, bacteriocin, etc.), production of antioxidants with protective effects against cell damage, and ability to inhibit inflammatory enzymes such as xanthine oxidase, cyclooxygenase, and lipoxygenase (Zhang et al. 2024). *T. ciferri*, however, is an opportunistic pathogen, identical to *Candida* species such as *C. tropicalis* and *C. famata*. As reported earlier, *C. tropicalis* and *C. famata* have been used as starter cultures after screening and confirmation of their nonpathogenicity (Banwo et al. 2021; Mancic et al. 2021; Pereira et al. 2022).

LAB and yeasts exhibit a mutual synergistic relationship during fermentation processes, wherein both organisms produce useful metabolites such as lactic acid, hydrogen peroxide, and diacetyl. The presence of yeasts in the fermenting matrix stimulates the growth of LAB (Umokaso et al. 2022). The identification of LAB and yeasts with technological qualities (i.e., production of amylase, lactic acid, diacetyl, and hydrogen peroxide) has been reported earlier by Fadahunsi and Soremekun (2017), Johansen et al. (2019), Guan et al. (2021), Banwo et al. (2022), and Akinyemi et al. (2022) in “Mahewu” (a South African fermented porridge), indigenous fermented foods and beverages in sub-Saharan Africa, *Lactobacillus*-fermented black barley, and raw Nigerian goat milk.

The identified yeasts and LAB strains tested positive for amylase production. Amylase production is a desired quality for the selection of yeasts and LAB as starter cultures, as these strains can use hydrolyzed starch for metabolic activities and increase the amount of metabolites produced during microbial fermentation, ultimately improving food quality (Nnawuihe et al. 2018; Adebiyi et al. 2019; Banwo et al. 2021). The production of lactic acid and hydrogen peroxide by LAB and yeasts supports previous claims that these species can be used as bio-control starters to inhibit undesirable organisms, including pathogenic organisms and aflatoxigenic fungi (Martinez-Villaluenga et al. 2017; Adebiyi et al. 2019).

Both LAB and yeast isolates in this study exhibited the capacity to synthesize lactic acid, diacetyl, and hydrogen peroxide. The synthesis of lactic acid and hydrogen peroxide by LAB and yeasts also strengthens their suitability for use as biopreservative starters that can inhibit the growth of undesirable organisms and extend food shelf-life. This observation aligns with the previous reports of Fadahunsi and Olubodun (2021) and Otunba et al. (2021). Diacetyl production by the identified yeasts and LAB further suggests their potential for use as flavoring agents in food production processes as diacetyl imparts a buttery aroma in foods, contributing to improved organoleptic properties. This observation agrees with the previous reports of Nasri et al. (2022) and Guo et al. (2023).

## Conclusions

*L. delbrueckii* RSL11, *Lactobacillus* sp. MLL5, *C. albidus* MZY30, *N. albida* WSY40, and *C. ciferri* MLY11 identified in the present study were confirmed to be nonpathogenic and to produce substantive amounts of antimicrobial metabolites; thus, these strains can be used as starter cultures for controlled fermentation processes to improve food organoleptic properties and extend the shelf-life of fermented foods. Lastly, the results obtained from this study indicate that LAB and yeasts are critical microorganisms with technological potentials, and are suitable for applications as food fermentation starter cultures to produce safe, and functional foods.
